# Supplementary material for: Integrated avalanche photodetectors for visible light
Source: Nat Commun. 2021 Mar 23;12:1834. doi: 10.1038/s41467-021-22046-x (PMC7988121; doi:10.1038/s41467-021-22046-x)
Supplement: Supplementary file 1 — Supplementary Information [file 41467_2021_22046_MOESM1_ESM.pdf]

Integrated Avalanche Photodetectors for Visible Light: Supplementary Information

Salih Yanikgonul,[1,2,‡] Victor Leong,[1,†] Jun Rong Ong,[3,*], Ting Hu,[4] Shawn Yohanes Siew,[5] Ching Eng Png,[3] and Leonid Krivitsky [1]

[1] *Institute of Materials Research and Engineering, Agency for Science, Technology and Research (A*STAR), 138634 Singapore*
[2] *School of Electrical and Electronic Engineering, Nanyang Technological University, 639798 Singapore*
[3] *Institute of High Performance Computing, Agency for Science, Technology and Research (A*STAR), 138632 Singapore*
[4] *Institute of Microelectronics, Agency for Science, Technology and Research (A*STAR), 138634 Singapore*
[5] *Advanced Micro Foundry, 117685 Singapore*
[‡] *Present address: Advanced Micro Foundry, 117685 Singapore*
[†] *victor_leong@imre.a-star.edu.sg*
[*] *ongjr@ihpc.a-star.edu.sg*

Supplementary Note 1. Coupling and Propagation Loss Measurements

We systematically characterized the coupling and propagation losses on our device by performing a series of cutback measurements with test waveguides. In addition to waveguide widths $W = 750$, 900 nm mentioned in the main paper, here we also investigated $W = 450$, 600 nm.

The optical transmission T through the device was obtained by measuring the input power P_A and output power P_B with a pair of lensed fibers (see Supplementary Figure 1(a)). Using SiN cutback waveguides of various lengths l_{SiN} (without the Si rib waveguide), we fitted our results using

$$T = P_B/P_A = \eta^2_{\text{f-SiN}}\, e^{-(\alpha_{SiN}\, l_{SiN})} \tag{S1}$$

to obtain the fiber-waveguide coupling loss $\eta_{\text{f-SiN}}$ and the SiN waveguide propagation loss coefficient α_{SiN}. A representative plot is shown in Supplementary Figure 1(b). Following this, we measured another series of devices that also included Si waveguides of various lengths l_{Si}; fitting our results to

$$T = P_B/P_A = \eta^2_{\text{f-SiN}}\, \eta^2_{\text{SiN-Si}}\, e^{-(\alpha_{SiN}\, l_{SiN})}\, e^{-(\alpha_{Si}\, l_{Si})} \tag{S2}$$

we obtained the SiN-Si end-fire coupling loss $\eta_{\text{SiN-Si}}$ and the Si waveguide propagation loss coefficient α_{Si}. The measured coupling and propagation losses are shown in Supplementary Figure 1(c). We note that we lack test structures for Si waveguides of width $W = 750$ nm; nonetheless we anticipate that the coupling and propagation losses will not significantly deviate from that of the other widths.

The observed fiber-waveguide coupling losses $\eta_{\text{f-SiN}}$ agree with our expected values. The slight increase in $\eta_{\text{f-SiN}}$ with width W is likely due to the larger inverse taper angle, since the taper length and tip width are kept constant for all widths W. The end-fire coupling loss $\eta_{\text{SiN-Si}}$ is ~3-4 dB larger than mode-matching calculations, which is attributed to fabrication imperfections resulting in a non-ideal waveguide interface. We also observed decreasing propagation losses with increasing width W.

For APD characterization, the total insertion loss into the active device structure (i.e. the Si rib waveguide) is given by

$$\eta_{total} = \eta_{\text{f-SiN}}\, \eta_{\text{SiN-Si}}\, e^{-(\alpha_{SiN}\, l_{SiN})} \tag{S3}$$

where $l_{SiN} = 0.3125$ cm is constant for all characterized devices. For both $W = 750$ nm and 900 nm, this yields $\eta_{total} = 7.1 \pm 0.4$ dB. We decided to focus on devices with lower insertion loss, and thus only considered devices with these two widths for further characterization in the main paper.

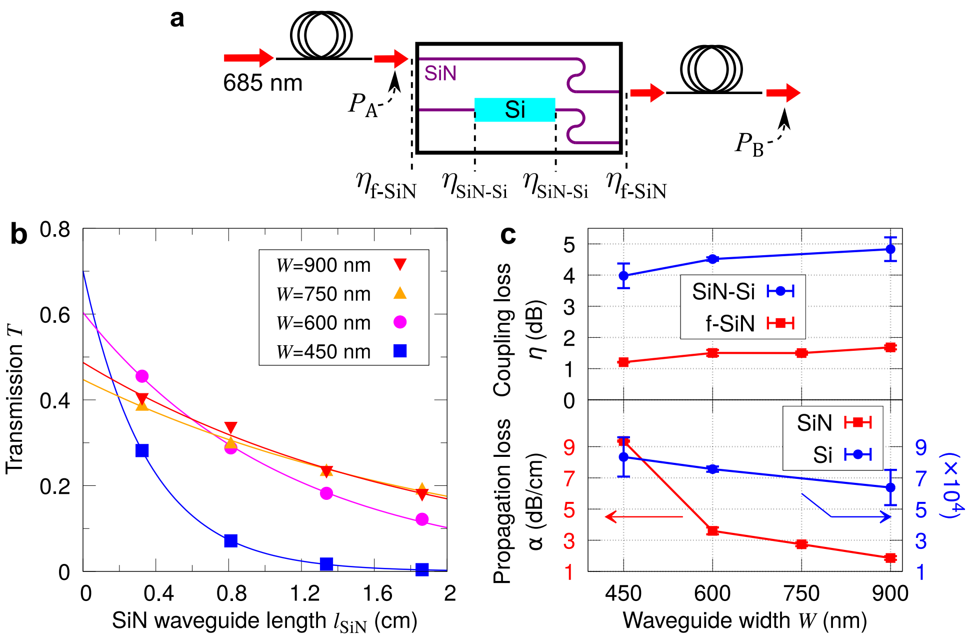

Supplementary Figure 1. **Optical coupling loss measurements.** (a) Schematic of the experimental setup, depicting the cutback waveguide structures and the various sources of coupling losses. Horizontally polarized 685 nm light is coupled to and from the waveguides via lensed fibers. The optical powers at both ends of the chip (denoted P_A and P_B) are measured. (b) Optical transmission measurements for SiN cutback waveguides of various lengths l_{SiN} and different waveguide widths W. The solid curves are exponential fits, see Eq. S1. (c) Measured coupling losses for different waveguide widths W. The results shown in (c) are the averaged measurements across several devices; error bars reflect the standard deviation.

Supplementary Note 2. Electro-optic characterization setup

The schematic of the electro-optic characterization setup is shown in Supplementary Figure 2. Detailed descriptions of the components are found in the Methods section of the main text.

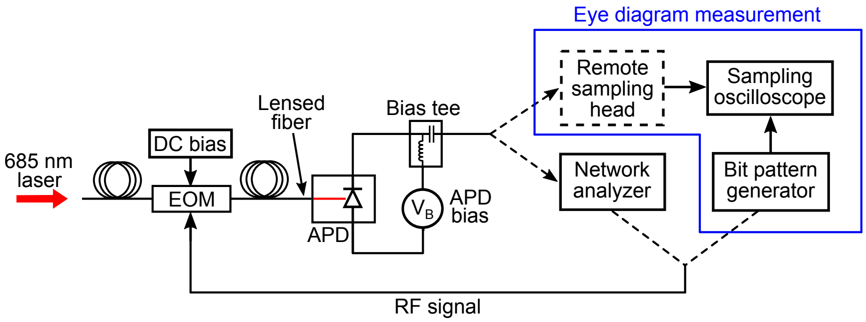

Supplementary Figure 2. **Schematic of the characterization setup.** Horizontally polarized (TE) 685 nm light, which can be modulated with an RF signal using an electro-optic modulator (EOM), is coupled to the on-chip SiN waveguide with a lensed fiber. Electrical connections to the devices are made via contact pads on the chip surface using electrical probes. A bias tee separates the AC and DC signals from the APDs. The AC signal is sent either to a network analyzer for bandwidth measurements, or to a sampling oscilloscope for eye diagram measurements. An additional remote sampling head was used at 56 Gbps to obtain a clearer signal.

Supplementary Note 3. Eye diagrams

The reference eye diagrams of the EOM output are shown in Supplementary Figure 3(a). We observe clear, open eyes at up to 56 Gbps, indicating that our measurement system performs well at these bit rates. We are unable to measure at higher bit rates due to our limitations in generating faster bit patterns.

In general, the signal-to-noise ratio (SNR) obtained from the eye diagram increases with the reverse bias V_B, as the signal amplitude increases with a larger gain. Supplementary Figure 3(b) shows the SNR increase with V_B for laterally doped devices at 25 Gbps.

The eye diagrams presented in Fig. 5 of the main text show the open eyes at the highest data rate measured for each device. Supplementary Figures 3(c),(d) show additional data obtained at different data rates for lateral and interdigitated devices, respectively. We observe that all devices can be operated at lower data rates with a higher SNR.

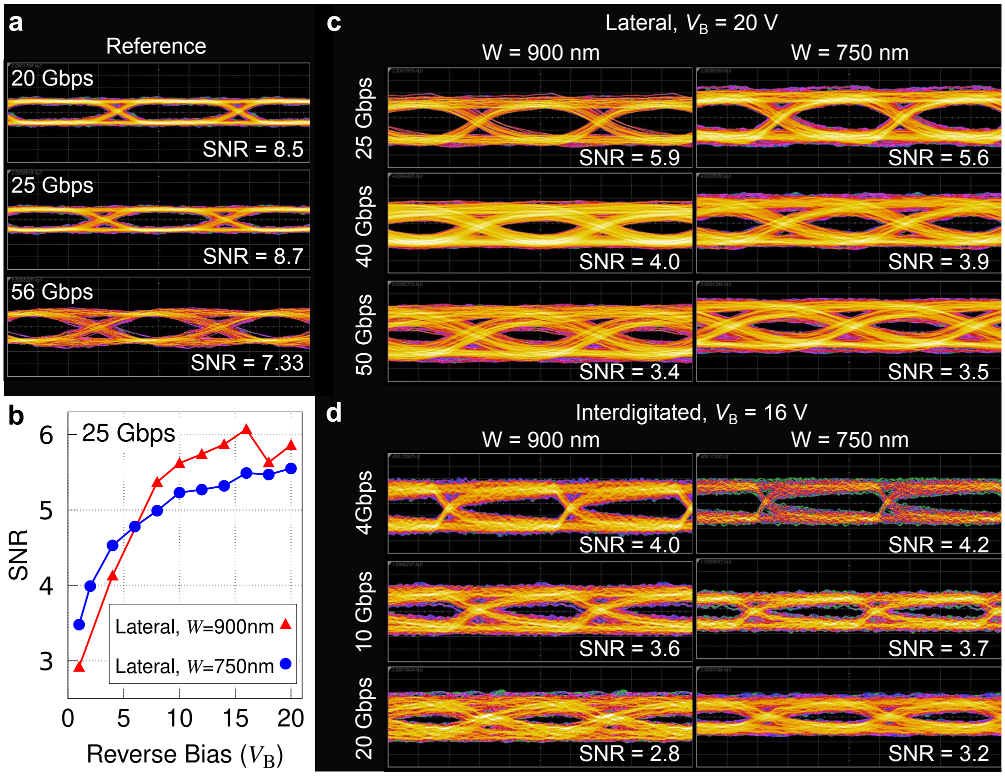

Supplementary Figure 3. **Additional eye diagram measurements**. (a) Reference eye diagrams of the EOM output at different data rates, measured with a Newport 1004 photodetector (3 dB bandwidth: 40 GHz) and the sampling oscilloscope. (b) Increase in signal-to-noise ratio (SNR) with the reverse bias V_B, obtained from eye diagrams measured for laterally doped devices. (c) Eye diagrams measured for laterally doped devices at $V_B = 20$ V at different data rates. (c) Eye diagrams measured for interdigitated devices at $V_B = 16$ V at different data rates.

Supplementary Note 4. Determination of the unity gain point

To determine the avalanche gain G at a particular reverse bias V_B, the measured photocurrent I_{ph} has to be compared to that measured at a low bias voltage V_{ug} where avalanche effects are negligible, i.e. the APD exhibits unity gain. We can then attribute any further increase in I_{ph} at $V_B > V_{ug}$ solely to the avalanche gain. An implicit assumption here is that the quantum efficiency (QE) — the efficiency of absorbing input photons and converting them into a photocurrent (without multiplication gain) — is saturated and remains essentially constant above V_{ug}.

However, at a low bias, the junction might not be fully depleted yet, and the QE might not have reached saturation. Thus, the increase of the photocurrent I_{ph} with V_B may be caused by both an increase in the QE and G, and it is difficult to distinguish between the two mechanisms. This leads to a difficulty in determining the unity gain bias V_{ug}.

In this section, we will present an empirical estimation based on the measured device photocurrent, as well as numerical simulations of the gain G and QE. Finally, we analyse our findings and conclude with our choice of V_{ug}.

Empirical estimation: 2nd derivative of the photocurrent with respect to bias

Some reports in the literature rely on a bias-independent photocurrent at low V_B to indicate unity gain [1,2]. However, we do not observe such a feature in our current-voltage measurements (see Fig. 2(a) of main text). Other reports assume full or nearly full depletion at low V_B [3,4], but it is not obvious that this assumption is valid for our devices: the increase in 3 dB bandwidth with bias up to $V_B \sim 10$ V suggests that the depletion region might still be widening.

Instead, we estimate the unity gain point by measuring where the second derivative of the photocurrent with respect to bias becomes zero [5,6], i.e. $\partial^2 I_{ph}/\partial {V_B}^2 = 0$. This yields a reasonable transition point between regimes where the increase in I_{ph} with V_B is likely dominated by a saturation in QE (at lower bias) and an increase in G (at higher bias). For an input optical power of $P_{opt} = 30.2 \pm 0.2$ dBm, we obtain V_{ug} of 1.45 – 1.7 V across all device types (see Supplementary Figure 4).

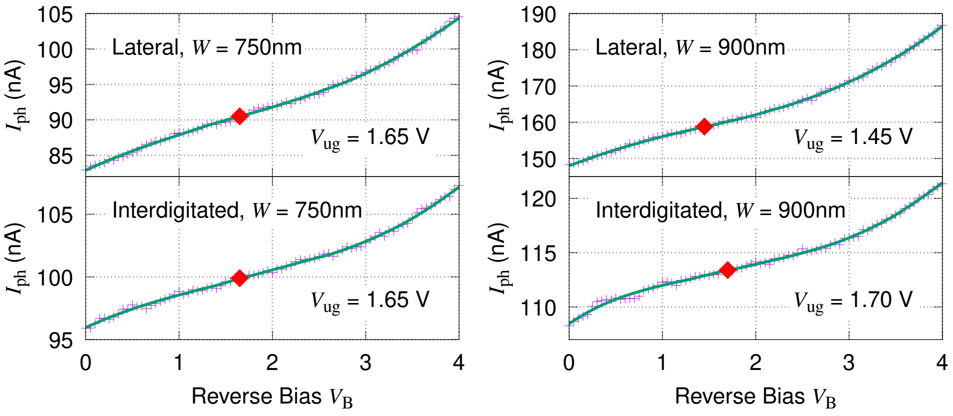

Supplementary Figure 4. **Empirical estimates of the unity gain point V_{ug}.** Each plot shows the measured photocurrent I_{ph} at an input power of $P_{opt} = 30.2 \pm 0.2$ dBm for a device type. From the data points, we obtain a smoothed curve (solid line), from which we calculate its second derivative with respect to V_B. The red diamonds mark the curves at V_{ug}, the reverse bias value where $\partial^2 I_{ph}/\partial {V_B}^2 = 0$.

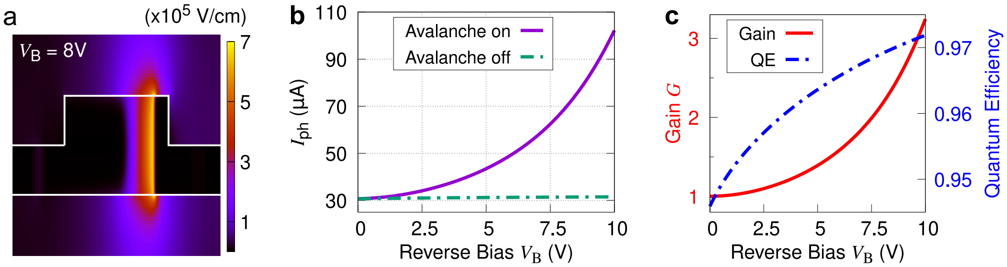

Supplementary Figure 5. **Simulations based on a laterally doped device with width $W = 900$ nm.** (a) Electric field at reverse bias $V_B = 8$ V. (b) Photocurrent I_{ph} with and without avalanche effects. (c) Avalanche gain G and quantum efficiency (QE).

Numerical simulations of gain and quantum efficiency

We simulate the DC electrical performance of laterally doped devices using the ATLAS device simulator (Silvaco Inc.), allowing us to analyze the electric field (an example is shown in Supplementary Figure 5(a)), ionization coefficients, charge carrier drift velocities, etc. Avalanche effects can be simulated by activating the impact ionization model (we choose the Selberherr's model) within ATLAS. By comparing the device photocurrent with and without the impact ionization model, we can extract a simulated value of the gain G (see Supplementary Figure 5(b),(c)). The gain increases smoothly with the bias V_B, and already starts to deviate from unity gain ($G = 1$) at low bias.

The QE can also be obtained by normalizing the simulated photocurrent to the input optical power (see Supplementary Figure 5(c)). While QE does vary with bias, it is already almost saturated at $V_B \sim 0$ V with a high QE of ~ 0.95. We note that the high doping concentrations lead to large built-in electric fields in the APD even without applied bias, which strongly accelerates the photogenerated charge carriers. Coupled with the small device size, this results in the charge carrier transit time being much shorter than the recombination lifetime. Thus, the photogenerated carriers are efficiently collected by the device before they are lost to recombination.

While it is sufficient to just simulate the 2D cross-section for laterally doped devices, interdigitated devices lack a convenient symmetry axis, and thus require full 3D simulations of the whole device. However, we do not have the required computational resources to perform a thorough quantitative analysis of the gain and QE for the interdigitated devices. Nonetheless, we assume the trends in gain and QE for the interdigitated devices will be similar to that of the lateral devices.

Analysis

Our empirical estimates of the unity gain point yield $V_{ug} \lesssim 2$ V. This is consistent with our simulation results, which show that QE is already high and that the gain G is already increasing even at these low bias voltages. Taking these into account, and to avoid overestimating the avalanche gain G and gain-bandwidth product (GBP), we conservatively consider the unity gain point to be $V_{ug} = 2$ V for all devices and input powers P_{opt} in our analysis.

Supplementary Note 5. Analysis of simulated electric field profiles

In this section, we will analyze the representative electric field profiles of both laterally doped and interdigitated devices of the same width W, and relate their features to the device characteristics we observe in our measurements. The electric field profiles are obtained using the ATLAS device simulator (Silvaco Inc.).

Supplementary Figure 6(a) shows the electric field profile in a laterally doped device, where the high-field regions are found along the p-n^+ junction within the waveguide core. More detailed simulation results of laterally doped devices can be found in our previous works [7, 8]. Supplementary Figure 6(b) shows the electric field profile of an interdigitated device. To reduce computation time, we limited the scale of the device to only two periods of alternating p-n^+ regions. Nonetheless, we are still able to obtain the necessary features for our analysis. Both figures are simulated at just above the breakdown voltage of the devices.

Peak electric field strengths

We observe that the highest electric field strengths in the interdigitated device are concentrated at the corners of the n^+-doped areas, and that their magnitude is significantly higher than that found in the laterally doped device with the same waveguide dimensions. The emergence of these localised high-field regions is likely to have resulted in a lower breakdown voltage V_{br} in interdigitated devices. This could also have contributed to the higher dark current observed in interdigitated devices, due to the exponential dependence of the dark carrier generation rate on higher field strengths.

Light absorption in undepleted n^+-doped regions

In a p-n^+ junction, the p-doped regions are fully depleted, but the depletion region only extends minimally into the n^+-doped regions due to their higher doping concentration. For the lateral doping profile, there is a large overlap between the depletion region and optical mode over the full length of the Si rib waveguide. However, for the interdigitated design, a significant amount of light absorption occurs in the undepleted n^+-doped regions, as input light propagates along the alternating p- and n^+-doped "digits".

It is less desirable for light absorption to occur in the undepleted n^+ regions. Due to the weak electric field strengths, the avalanche multiplication of the photo-generated charge carriers

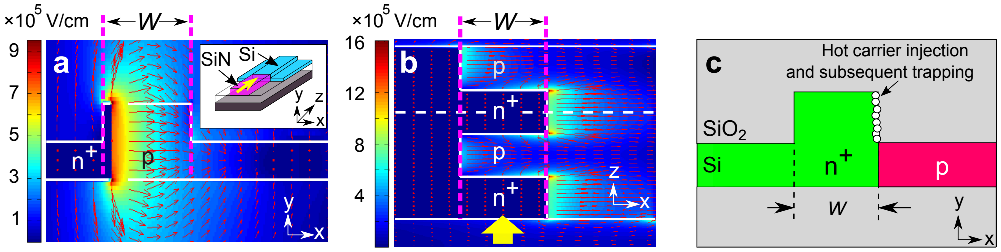

Supplementary Figure 6. **Simulated electric field profiles.** (a) Electric field profile of a laterally doped device. Inset shows the schematic of the APD structure (top cladding omitted for clarity) and the axis orientations. (b) Electric field profile of an interdigitated device. The highest electric field strengths are concentrated at the corners of the n^+-doped areas. The devices in both (a) and (b) have the same width W, and are simulated at just above the breakdown voltage. The yellow arrows indicate the propagation direction of input light. (c) Schematic of the waveguide cross-section along the horizontal white dashed line in (b). We mark the interface where we expect significant injection and subsequent trapping of hot carriers.

is less efficient compared to the high-field depletion region, and thus it is detrimental to the overall device gain and responsivity. This might explain the slightly lower responsivity observed in $W = 900\,\text{nm}$ interdigitated devices compared to laterally doped devices, though we do not observe a significant difference for $W = 750\,\text{nm}$ devices. The slower charge carrier diffusion in the low-field regions [7] would also contribute to the lower device bandwidth observed in our interdigitated devices.

In our devices, input light is first incident on a n^+-doped region. This results in an overall slightly higher absorption (a difference of ~10%) in n^+-doped compared to p-doped regions. Thus, the effect of light absorption in the n^+-doped regions could be slightly reduced by having input light incident on the opposite end of the Si waveguide, such that the light is first incident on a p-doped region.

The dimensions of the interdigitated doping regions can potentially be optimized, e.g. the pitch and length of each doping region, or to have p- and n^+-doped regions of different lengths. However, we foresee a design trade-off as increasing the depletion volume would also likely increase the device capacitance, which could lead to an RC-limited bandwidth.

Charge trapping

Charge trapping can occur as hot carriers are injected into the SiO_2 cladding, and are subsequently trapped at the interface between the n^+-doped region and the cladding. The trapped charges would change the electrical field distribution inside the depletion region over time [9]. This effect is likely more severe in interdigitated devices, as the high electric fields occur at the edge of the Si rib waveguide (see Supplementary Figure 6(c)). This could lead to drifts in breakdown voltage and device gain over time, which is discussed in detail in the next section.

A potential mitigating strategy is to include guard-ring structures [10] at the Si-SiO_2 interface. In addition, adopting a shallow etch for the Si rib waveguide would also reduce the interface area for charge trapping.

Supplementary Note 6. Decaying gain and breakdown voltage drifts at high bias

In our devices, the device breakdown voltage V_{br} drifts towards higher values over time as a reverse bias voltage V_B is continuously applied. This is accompanied by an observed decay in the photocurrent and dark current from the onset of applying the reverse bias. Representative measurements based on a $W = 900$ nm laterally doped device are shown in Supplementary Figure 7(a),(b). The decrease is more pronounced at higher V_B, with a steep decay in the current at the start before gradually leveling off; while at lower V_B the decay is much slower.

There is a corresponding decrease in the gain G with time, as shown in Supplementary Figure 7(c). While the effect is minimal at lower V_B, where G decreases by <10% over 10 mins for V_B <13 V at P_{opt} = -25 dBm, the drop in gain increases sharply at high V_B. The rate of decrease slows down significantly after the first 10 mins, but full stability of G is observed only after ∼ 30 mins.

The V_{br} drift has been reported in other APDs [11, 12]. As discussed above, this effect is likely to be more severe in interdigitated devices. This is consistent with our observation of a larger gain reduction over time for interdigitated devices (comparing Table 1 and Fig. 4 of the main text).

This phenomenon reveals two distinct operating modes for our devices: a gated mode where the APD is operated at high V_B with high gain, using a reset procedure to circumvent the decay in gain (explained in the following section); and a continuous mode where the APD is either operated at low V_B, or after the gain has stabilized over some time under a higher V_B.

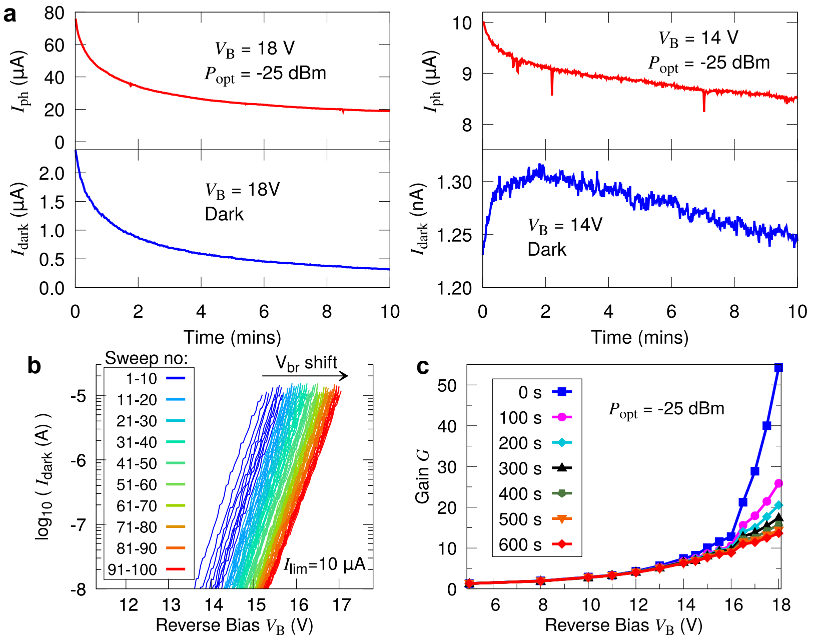

Supplementary Figure 7. **Decaying gain and breakdown voltage drifts for a $W = 900$ nm laterally doped device.** (a) Photocurrent I_{ph} (measured at P_{opt} = -25 dBm) and dark current I_{dark} at reverse bias V_B of 14 V and 18 V. Prior to each measurement, the device is reset with the application of a forward bias voltage. (b) The avalanche breakdown voltage V_{br} increases upon successive voltage sweeps. Each sweep starts from $V_B = 0$ V and is terminated upon the dark current I_{dark} reaching the breakdown current of 10 µA. Here, the device is not reset with a forward bias voltage in between runs. (c) Change in gain G over time at different V_B. Here, the reverse bias is continuously applied.

Supplementary Note 7. Reset procedure for gated operation with high gain

Despite the APD gain decreasing over time, it can be reset by the application of a forward bias voltage V_F. This likely causes the de-trapping of the charge carriers, allowing the device gain to recover to its original value. The procedure is illustrated in Supplementary Figure 8(a). In between the sweeps of the reverse bias V_B used to characterize the APD, we apply $V_F = -1$ V for 1 s to the device cathode; a shorter duration might be sufficient, but we did not investigate this in detail. We note that our measurement instrument limitations result in a delay of ~1 s when switching between V_B and V_F.

The reset procedure prevents the drift in the breakdown voltage over successive voltage sweeps (see Supplementary Figure 8(b)), and also results in repeatable current-voltage characteristics after each reset. For the DC characterization results presented in the main report, the reset procedure is carried out before each measurement.

Thus, we are able to periodically operate the APD in the high-gain regime with a gated mode, which would be compatible with applications where gating is used to reduce noise and enhance the signal. Such applications include time-of-flight imaging [13], low-light imaging [14], and Raman spectroscopy [15]. We also note that Geiger-mode infrared InGaAs APDs often employ gating techniques to suppress dark counts and afterpulsing [16].

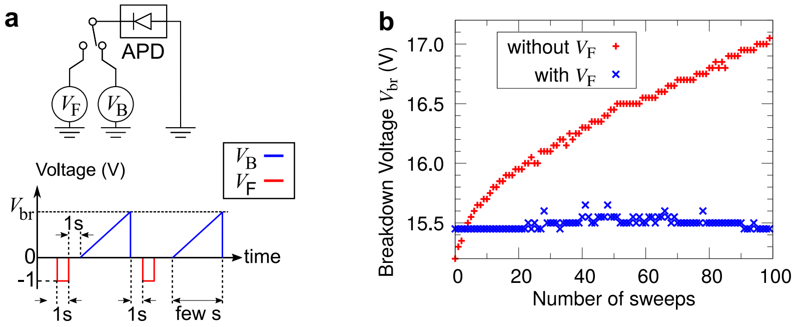

Supplementary Figure 8. **Reset procedure for high-gain operation.** (a) Schematic of the procedure. Here, multiple sweeps of the reverse bias voltage V_B are applied to the APD. Prior to each sweep, a forward bias voltage of $V_F = -1$V is applied to the APD cathode for 1 s. The time axis is not drawn to scale. (b) Comparing breakdown voltage V_{br} drifts for a $W = 900$ nm laterally doped device. By applying the forward bias V_F, the breakdown voltage V_{br} remains stable over successive sweeps of V_B. If V_F is not applied, we obtain the same results in Supplementary Figure 7(b).

Supplementary References

1. Virot, L. *et al.* Germanium avalanche receiver for low power interconnects. *Nature Communications* **5**, 4957 (2014).
2. Chen, H. T. *et al.* High sensitivity 10Gb/s Si photonic receiver based on a low-voltage waveguide-coupled Ge avalanche photodetector. *Optics Express* **23**, 815 (2015).
3. Zhu, H., Goi, K. & Ogawa, K. All-silicon waveguide photodetection for low-bias power monitoring and 20-km 28-Gb/s NRZ-OOK signal transmission. *IEEE Journal of Selected Topics in Quantum Electronics* **24**, 1–7 (2018).
4. Chen, H. T. *et al.* 25-Gb/s 1310-nm optical receiver based on a Sub-5-V waveguide-coupled germanium avalanche photodiode. *IEEE Photonics Journal* **7**, 1–9 (2015).
5. Meier, H. T. J. *Design, characterization and simulation of avalanche photodiodes*. Ph.D. thesis, ETH Zurich (2011).
6. Lumerical. Avalanche photodetector. https://support.lumerical.com/hc/en-us/articles/360042454814-Avalanche-photodetector. Accessed: 2020-12-24.
7. Yanikgonul, S., Leong, V., Ong, J. R., Png, C. E. & Krivitsky, L. 2D monte carlo simulation of a silicon waveguide-based single-photon avalanche diode for visible wavelengths. *Optics Express* **26**, 15232–15246 (2018).
8. Yanikgonul, S., Leong, V., Ong, J. R., Png, C. E. & Krivitsky, L. Simulation of silicon waveguide single-photon avalanche detectors for integrated quantum photonics. *IEEE Journal of Selected Topics in Quantum Electronics* **26**, 1–8 (2019).
9. Liu, S. *et al.* Repetitive-avalanche-induced electrical parameters shift for 4H-SiC junction barrier schottky diode. *IEEE Transactions on Electron Devices* **62**, 601–605 (2015).
10. Lee, M.-J., Rucker, H. & Choi, W.-Y. Effects of guard-ring structures on the performance of silicon avalanche photodetectors fabricated with standard CMOS technology. *IEEE Electron Device Letters* **33**, 80–82 (2011).
11. Verwey, J., Heringa, A., de Werdt, R. & v.d. Hofstad, W. Drift of the breakdown voltage in p-n junctions in silicon (walk-out). *Solid-State Electronics* **20**, 689–695 (1977).
12. Neugebauer, C., Burgess, J., Joynson, R. & Mundy, J. Electron trapping in thin SiO2 films due to avalanche currents. *Thin Solid Films* **13**, 5–9 (1972).
13. Morimoto, K. *et al.* Megapixel time-gated SPAD image sensor for 2D and 3D imaging applications. *Optica* **7**, 346–354 (2020).
14. Resetar, T. *et al.* Development of gated pinned avalanche photodiode pixels for high-speed low-light imaging. *Sensors* **16**, 1294 (2016).
15. Kostamovaara, J. *et al.* Fluorescence suppression in raman spectroscopy using a time-gated CMOS SPAD. *Optics Express* **21**, 31632–31645 (2013).
16. Liang, Y., Fei, Q., Liu, Z., Huang, K. & Zeng, H. Low-noise InGaAs/InP single-photon detector with widely tunable repetition rates. *Photonics Research* **7**, A1–A6 (2019).
